# Targeting postoperative sleep disorders: Current advances and emerging directions in stellate ganglion block therapy

**DOI:** 10.1097/MD.0000000000043256

**Published:** 2025-07-18

**Authors:** Jianghui Luo, Xudong Zhang, Xiaohong Tan, Jiazun Chen, Miaomiao Tang

**Affiliations:** a Department of Anesthesiology, Sichuan Cancer Hospital and Institute, Sichuan Cancer Center, School of Medicine, University of Electronic Science and Technology of China, Chengdu, Sichuan, China.

**Keywords:** anesthesia, pain, postoperative sleep disorder, stellate ganglion block, surgical procedure

## Abstract

The stellate ganglion block (SGB) is an extensively utilized regional anesthesia technique, integral to pain management protocols and postoperative analgesia. Its fundamental objective lies in the modulation of sympathetic nervous system activity, thereby facilitating the intricate balance of the autonomic, circulatory, endocrine, and immune systems to preserve homeostatic equilibrium. Postoperative sleep disorders (PSD), characterized by disruptions in sleep quantity and quality subsequent to surgical intervention, manifest as oscillations in the sleep–wake cycle and are indicative of postoperative neurocognitive impairments. Emerging research underscores the efficacy of SGB in mitigating PSD incidence through the targeted regulation of sympathetic ganglionic excitability and the modulation of endocrine and autonomic nervous system functions. This study offers an exhaustive appraisal of the effects of SGB on PSD, delving into the anatomical foundations of the procedure, elucidating its physiological mechanisms, and amalgamating pivotal insights derived from recent scholarly research.

## 1. Introduction

The stellate ganglion (SG) is a complex structure formed by the fusion of the 6th (C6) and 7th cervical (C7) ganglia, along with the first thoracic (T1) ganglion.^[1,2]^ It plays a crucial role in the autonomic nervous system, containing sympathetic preganglionic fibers that innervate the head and neck as well as sympathetic postganglionic fibers responsible for innervating the upper limbs and the heart. Stellate ganglion block (SGB) is a minimally invasive therapeutic technique widely used in the fields of pain medicine and anesthesia.^[3,4]^ This procedure involves the precise injection of local anesthetics into the vicinity of the SG, pre- and postganglionic nerves, and their corresponding innervated regions. By doing so, it effectively interrupts sympathetic nerve transmission to the head, face, neck, shoulder, upper limbs, anterior chest, and posterior back, leading to the modulation of sympathetic nervous system tons. Ultimately, this modulation extends to the nervous, circulatory, endocrine, and immune systems to maintain dynamic balance.^[1,5,6]^ Currently, SGB enjoys widespread utilization across various clinical domains, demonstrating remarkable efficacy not only in managing painful conditions but also in the treatment of non-painful diseases.^[3,7–9]^ This article employs the bibliometric method to explore the extensive use SGB in clinical settings and animal research, primarily delving into the research advancement of SGB in enhancing postoperative sleep. The examination is conducted from anatomical, mechanistic, and functional perspectives, providing a comprehensive overview of the subject.

## 2. Examining a decade of SGB research: a bibliometric analysis

**Figure 1.**
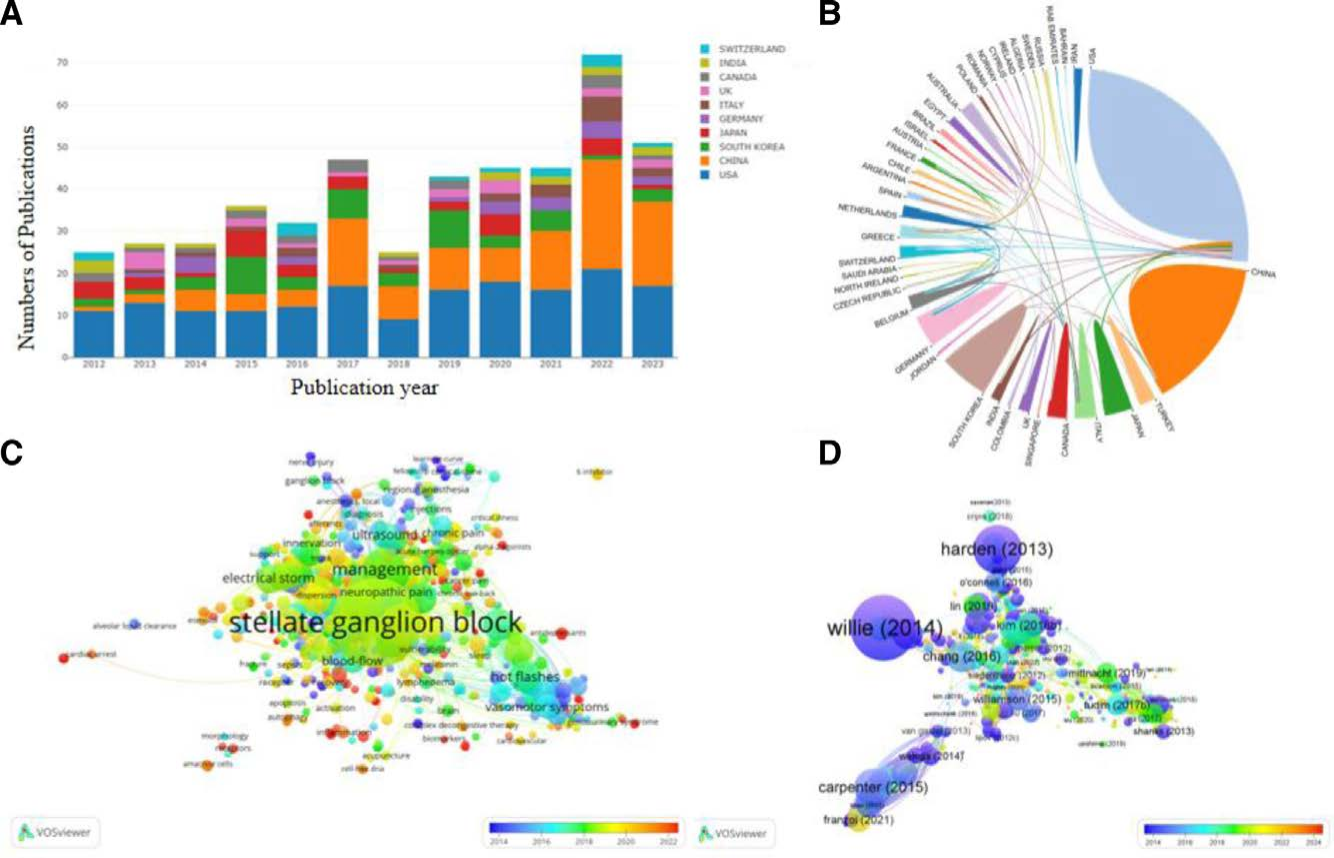
Bibliometric analysis in stellate ganglion block from 2012 to 2023. (A) Trend analysis of stellate ganglion block publications (2012–2023), there has been a rapid surge in the number of publications related to stellate ganglion block, reaching its apex in 2022. The countries at the forefront of this research surge include China, the US and South Korea, with Japan, Germany, Italy, UK, Canada, India and Switzerland also contributing significantly. This sharp uptick in annual publications across various nations underscores the growing global interest and dedication of researchers to stellate ganglion block. (B) Mapping national collaborative networks, the period from 2012 to 2023 has been the formation and strengthening of national cooperative relations networks, illustrating the collaborative efforts worldwide to advance stellate ganglion block. (C) Key word proportion analysis, an in-depth analysis of the keyword proportions related to stellate ganglion block from 2012 to 2023 provides valuable insights into the evolving focus areas and terminologies prevalent in this field of study. (D) Examination of highly cited reference, a comprehensive analysis of the most cited references in stellate ganglion block studies between 2012 and 2023 offers a glimpse into the seminal works and influential research that have shaped the current understanding of this technique.

Over the past decade, the use of SGB in research has seen significant growth. This is evident from an analysis of 1032 articles retrieved from the core collection of the Web of Science database. The comprehensive bibliometric analysis covered various dimensions, including annual publication trends, leading journals in the field, highly cited papers, and prevalent keywords. To conduct this investigation, we utilized tools such as a specialized bibliometric analysis platform (http://bibliometric.com) and VOS viewer software, enabling us to scrutinize the subject words present in the compiled literature. The limitation of these bibliometric software is that it can only analyze the data from the web of science database. Our findings indicate a sharp uptick in SGB-related studies beginning in 2012, as depicted in Fig. 1A. In terms of geographical contributions, China, the USA, and South Korea emerged as the top 3 nations publishing on this topic, with Fig. 1B illustrating a close-knit cooperation network amongst them. A review of the last 10 years of publications reveals that journals “Pain physician” hosted the majority of SGB studies, followed closely by “Pain medicine” and “Regional Anesthesia & Pain medicine” (Table 1). Furthermore, an analysis of keyword evolution provides insights into the prevailing trends and future directions of SGB research, as showcased in Fig. 1C and D.

**Table 1:** Top 10 journals for stellate ganglion block research published between 2012 to 2023.

Journal	Impact factor (2023)	Total numbers of publications	Country	Total numbers of citation
Pain Physician	3.7	15	United states	95
Pain Medicine	3.1	20	United states	92
Regional Anesthesia & Pain Medicine	5.1	17	United states	83
Journal of Cardiovascular Electrophysiology	2.7	8	United states	67
Military medicine	1.2	7	United states	63
Current Pain and Headache Reports	3.7	4	United states	51
The Journal of the North American Menopause Society	2.7	7	United states	39
Journal of Clinical Anesthesia	6.7	8	United states	37
Jama Psychiatry	25.8	5	United states	34
Arrhythmia & Electrophysiology Review	3	4	United Kingdom	32

## 3. Anatomy of SG

**Figure 2.**
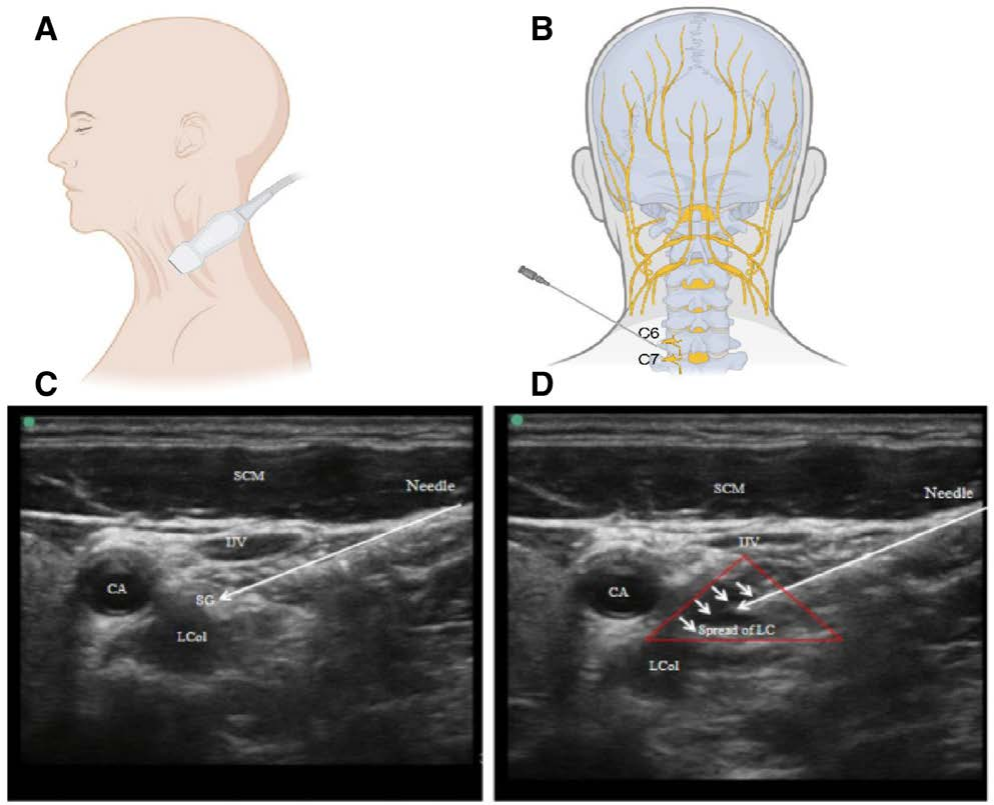
The anatomy and ultrasound image of the stellate ganglion block. (A and B) Anatomy structure and puncture point of stellate ganglion block; (C and D) the ultrasound image of the stellate ganglion block. CA = carotid artery, IJV = internal jungle vein, LC = local anesthetic, Lcol = Longus colli muscle, SCM = sternocleidomastoid muscle.

Located on both sides of the human neck, behind the carotid sheath and in front of the cervical vertebral transverse processes, are 3 cervical sympathetic ganglia, namely the superior cervical ganglion, middle cervical ganglion, and inferior cervical ganglion.^[1,10,11]^ The SG is specifically situated in front of the base of the C7 and the first rib neck, adjacent to the carotid sheath in the anterior aspect. Internally, it is adjacent to the longus colli muscle and posteriorly connected to the intervertebral foramen and recurrent laryngeal nerve. Externally, it neighbors the anterior scalene muscle and phrenic nerve. Anterolaterally, it is connected to the brachiocephalic vein and the thyroid cervical trunk. Below, it borders the apex of the lung and the pleura, belonging to the inferior cervical ganglion.^[5]^ In 60% to 80% of the population, the inferior cervical ganglion fuses with the T1 ganglion, but sometimes it may also include the second thoracic (T2) ganglion and middle cervical ganglion. This amalgamation is enveloped by surrounding connective tissue and adipose tissue, forming the cervicothoracic ganglion.^[5,11]^ The morphology of the cervicothoracic ganglion is irregular, typically star-shaped(66. 67% on the left, 33. 33% on the right), hence it is also referred to as SG.^[12]^ The size of SG is approximately 25 mm × 10 mm × 5 mm,^[5,6,11]^ with its central position usually located at the level of the first rib neck on the top of the pleura. When the inferior cervical ganglion does not fuse with the T1 ganglion, it is usually located in front of the C7 transverse process, while the T1 ganglion is positioned in front of the first rib neck.^[11]^ The SG receives preganglionic sympathetic nerve fibers from the upper thoracic segments along the sympathetic chain, where they synapse with postganglionic neurons, giving rise to postganglionic nerve fibers. These fibers innervate multiple organ tissues, including the head, face, neck, upper limbs, and heart, as well as the major blood vessels, lungs, bronchi, and chest wall. In most cases, the lower border on the SG is situated above the upper margin of the second rib, with the pleura covering it below. There is typically a layer of adipose tissue covering it, which is often used as a marker for confirming the position of the SG.^[11]^ Based on the relationship between the lower border of the ganglion and the upper margin of the second rib, the occurrence rate of the SG being lower than the upper margin of the second rib is 33.3%. However, there have been no reports of the lower border of the SG being lower than the midpoint between the upper and lower margins of the second rib. Precisely locating the position of the SG and determining the puncture point and direction are crucial for performing nerve blockade. Structures such as the transverse process of C6 and C7, the carotid tubercle, the posterior midpoint of the sternocleidomastoid muscle, the inner edge of the jugular vein notch, and the sternoclavicular joint, which are bony or muscular, can all be used as surface landmarks to confirm the position of the SG^[13]^ (Fig. 2). Among these, the C7 transverse process is the closest to the SG and is the optimal bony landmark for projecting the SG onto the surface. However, it is important to note that the C7 transverse process lacks anterior tubercle, and the vertebral artery is not protected by the vertebral foramen, making it vulnerable to injury during puncture at this position^[13]^ (Fig. 2).

## 4. The wide application of SGB in clinical practice

Functionally, the SG belongs to the sympathetic ganglion, and it exerts a predominant influence when the body is under tension. It extensively affects the cardiovascular system and other visceral organs. Stimulation of the sympathetic nervous system can lead to several physiological responses, including the constriction of peripheral blood vessels and visceral blood vessels in the abdomen, reduced secretion of the digestive gland, dilation of pupils, and an increase in heart rates.^[5,6,13]^ The effects of SGB can be mainly divided into central and peripheral aspects. In its central aspect, SGB primarily acts on the hypothalamus to regulate the autonomic nervous system. In the peripheral aspect, it mainly affects the sympathetic preganglionic and postganglionic fibers. The combined effects of these 2 aspects result in the suppression of glands, blood vessels, and muscle activity in the innervated areas. The gastrointestinal tract is primarily controlled by both the central nervous system and the enteric nervous system.^[9]^ Previous studies have found that SGB can increase blood flow in the stomach and promote gastric motility, thereby aiding in the recovery of patients.^[9,14]^ This is because SGB can stimulate the parasympathetic vagus nerve in the stomach by modulating the hypothalamus, inhibiting the sympathetic nervous system, and promoting early gastrointestinal function recovery.^[9]^ Furthermore, a substantial body of literature reports on the favorable clinical efficacy of SGB in the treatment of conditions associated with sympathetic-parasympathetic imbalance. These conditions included headaches, postoperative sleep disorders (PSD), premenstrual syndrome, and more.^[4,8,15–17]^ Research has demonstrated a close relationship between SGB and the cardiovascular system, with SGB affecting various aspects such as heart rate, cardiac atrioventricular conduction, myocardial contractility, sinus rhythm, coronary blood supply, cardiovascular hormonal regulation, blood pressure, and peripheral tissue blood supply.^[5,6,18–20]^ Blocking the right SG can significantly inhibit cardiac sympathetic activity and the function of cardiopulmonary pressure receptors without a significant impact on blood pressure. However, when both sides are blocked simultaneously, it leads to a substantial decrease in heart rate and blood pressure.^[20]^ As a result, it is essential to avoid simultaneous bilateral SGB in clinical applications to mitigate risks to the cardiovascular system. Furthermore, the regulatory effect of SGB on the immune system should not be underestimated.^[21]^ A study found that SGB can enhance immune function and increase the survival rate by improving the function of splenic CD4-+-T cells after hemorrhagic shock.^[22]^ During the perioperative period, anesthesia and surgery induce sympathetic excitation, affecting the sensitivity and excitability of sensory neurons. This leads to vasoconstriction, local organ ischemia and hypoxia. It also triggers the release of neurotransmitters like norepinephrine (NE) and substance P, further constricting blood vessels and exacerbating local organ ischemia and hypoxia. This creates a vicious cycle, resulting in inflammation and pain.^[23]^ SGB can block the reflex pathway of the spinal cord, reduce sympathetic excitability and sensitivity, and decrease the release of harmful substances and inflammatory factors, thereby reducing pain. Animal studies also suggest that the analgesic effect of SGB may be achieved by reducing the release of spinal cord substance P and peripheral plasma catecholamines.^[24]^ Additionally, SGB has been shown to reduce the upregulation of thalamic hypoxia-inducible factor-1 alpha and NOD-like receptor thermal protein domain-associated protein 3 signaling. It also mitigates the hyperactivation of microglia and astrocytes, as well as the levels of inflammatory cytokines and oxidative stress. Furthermore, repeated SGB has been demonstrated to prevent central poststroke pain induced by thalamic hemorrhagic stroke, along with comorbid anxiety and depression.^[25]^ Over the past decades, SGB has also been used to treat post-traumatic stress disorder (PTSD).^[7,26]^ Study has reported that patients with PTSD who receive consecutive SGB treatment experience a significant decrease in Clinician-Administered PTSD Scale-5 scores with no related side effects.^[7]^

## 5. SGB treat for PSD

In recent years, clinical studies focusing on the use of SGB to improve PSD have garnered increasing attention^[14,27–35]^ (Table 2). PSD refers to abnormalities in postoperative sleep quantity or quality that result in a disrupted sleep–wake cycle, which is a manifestation of postoperative brain dysfunction.^[36]^ Patients typically experience a significant decline in sleep quality within the first 24 hours postoperatively, including a noticeable reduction in total sleep duration, slow wave sleep duration, and rapid eye moment sleep (REM) duration, with an increase in the number of awakenings.^[28,34]^ PSD causes significant adverse effects on patients’ postoperative recovery, contributing to issues such as cognitive dysfunction, mental confusion, pain sensitization, and an increased risk of cardiovascular events. These complications often result in prolonged hospitalization and a higher mortality rate.^[37]^ A previous study reported an incidence rate of PSD of approximately 42%. Among these cases, around 23% of patients continue to experience persistent PSD up to the 4th postoperative day, and roughly 25% report sleep disturbance on the 15th day postoperatively. In fact, 24% of these patients require medication to manage their sleep-related issue.^[37]^ Currently, interventions for PSD often involve the use of benzodiazepines or drugs like dexmedetomidine, sometimes antidepressants, and antianxiety medications. These medications induce passive sleep-in patients by inhibiting central nervous system activity. However, long-term use of these medications can lead to psychological and physiological dependence and may have various side effects. Previous study has shown that SGB can effectively block cervical sympathetic ganglia, leading to the dilation of brain blood vessels, improved cerebral blood flow, and regulation of cerebral microcirculation. This, in turn, alleviates sleep disorders.^[38]^ Several investigations have explored the use of SGB in the treatment of sleep disturbances after breast cancer surgery and found that SGB can reduce the consumption of anesthetics during surgery, decrease the incidence of postoperative complications, and result in higher patients’ satisfaction scores.^[27,28,31–33,35]^ Additionally, SGB has been shown to improve postoperative sleep quality, promote gastrointestinal function recovery, enhance postoperative recovery quality, and be free of associated side effects. One study even found that SGB produces similar effects to paroxetine in improving sleep and reducing hot flashes in breast cancer patients without the drug-related side effects.^[31]^ Similarly, a clinical study demonstrated that the combination of SGB and pectoral nerve block effectively reduces stress response and acute postoperative pain in patients undergoing radical breast cancer surgery while maintaining good hemodynamic stability during the perioperative period and improving postoperative sleep quality.^[35]^

**Table 2:** Examples of applications of the stellate ganglion block for various surgical procedures on sleep quality.

Study	Region	No. patients	Age	Background anesthesia	Methods of SGB	Control arm	Local anesthetics use in SGB	Success of SGB	Surgery	Findings	Study design
Yang et al^[27]^	China	30 versus 30	51.4 ± 6.5 versus 50.3 ± 6.8	GA	USG right-sided single injection SGB	USG right-sided single injection SGB with 6 mL saline	6 mL 0.25% ropivacaine	Horner syndrome	Breast cancer surgery	①SGB improve the QoR-40 in patients undergoing breast cancer surgery; ②Less intraoperatively need for propofol ③ Better postoperative recovery of sleep and gastrointestinal function	RCT
Yang et al^[28]^	China	25 versus 23	47.72 ± 7.90 versus 48.61 ± 8.19	GA	USG right-sided single injection SGB	Just an ultrasound scan	3 mL of 0.5% ropivacaine	Horner syndrome	Breast cancer surgery	SGB improves postoperative sleep quality and analgesia breast cancer surgery patients.	RCT
Yan et al^[14]^	China	20 versus 20	69.5 ± 3.98 versus 69.90 ± 4.53	GA + EA	USG left-side single injection SGB	GA + EA	5 mL of 0.375% ropivacaine	Horner syndrome	Gastrointestinal radical surgery	SGB alleviates POSD by reducing postoperative inflammatory response, increasing melatonin levels, and stabilizing perioperative hemodynamics	RCT
Xu et al^[29]^	China	1	30	–	USG alternating between right and left side SGB	–	5 mL 0.16% ropivacaine	Horner syndrome	-	SGB resulted in attractive effects regarding EDS recovery	Case report
Wu et al^[30]^	China	43 versus 44	61.4 ± 8.9 versus 59.6 ± 7.8	GA	USG single injection SGB	Just an ultrasound scan	0.5% 5 mL ropivacaine	–	Thoracoscopic surgery	SGB effectively improves the objective sleep quality	RCT
Poupak et al^[31]^	iran	20 versus 20	34.80 ± 5.317 versus 33.85 ± 5.566	Midazolam 1 mg + fentanyl 50 µg	USG single injection SGB	7.5 mg of paroxetine	10 mL of 0.5% bupivacaine	Horner syndrome	Breast cancer	SGB is as much effective as paroxetine in controlling hot flashes and sleep disturbances in breast cancer survivors	Clinical trial
Lipov et al^[32]^	USA	13	38–71	-	USG single injection right-side SGB	–	7 mL of 0·5% bupivacaine	Horner syndrome	Breast cancer survivors	①SGB can relief the sleep dysfunction breast cancer survivors ②SGB improves overall quality of life	Pilot study
Haest et al^[33]^	Belgium	34	34–69	–	A maximum of 3 blocks was permitted	–	10 mL of levobupivacaine 0.25%	Horner syndrome	Breast cancer survivors	Sleep quality was maintained out to 24 weeks the efficacy of SGB for hot flashes was reduced over time	Pilot study
Gu et al^[34]^	China	36 versus 35	68.01 ± 4.2 versus 67.97 ± 4.3	GA	USG single injection right-side SGB	USG right-sided single injection SGB with 7 mL saline	0.5% ropivacaine 7 mL	Horner syndrome	Thoracoscopic surgery	①SGB improves objective and subjective sleep quality ②SGB alleviate stress responses and sleep disorders, reduce postoperative hospital stay, and accelerate postoperative recovery of the patients.	RCT
Geng et al^[35]^	China	25 versus 25	54.52 ± 7.81 versus 55.76 ± 9.59	GA	① USG single injection right-sided SGB ② PNB	PNB with 20 mL of 0.375% ropivacaine	① SGB with 5 mL of 0.15% ropivacaine ② PNB with 20 mL of 0.375% ropivacaine	Horner syndrome	Modified radical mastectomy	PNB combined with SGB block effectively alleviate stress response and postoperative acute pain with stable perioperative hemodynamics and better postoperative sleep quality	RCT

EA = epidural analgesia, EDS = excessive daytime sleepiness, GA = general anesthesia, Horner syndrome = fascial flush, enophthalmos, ptosis, miosis, and conjunctival congestion, PNB = pectoral nerve block, QoR-40 = quality of recovery, RCT = randomized controlled trial, SGB = stellate ganglion block, USG = ultrasound-guided.

In a clinical study focusing on gastrointestinal tumors, researchers discovered that SGB effectively alleviates PSD by reducing postoperative inflammation, increasing melatonin levels, and maintaining hemodynamic stability during the perioperative period.^[28]^ Similar findings were validated in 2 separate thoracic surgery studies, revealing that SGB can improve both objective and subjective sleep quality, reduce stress responses, and PSD while also shortening postoperative hospitalization time and promoting patients’ postoperative recovery.^[30,34]^ Furthermore, previous studies have reported that SGB not only improves perioperative PSD but also significantly enhances the long-term sleep quality of patients experiencing PSD patients.^[31,32]^

## 6. The potential mechanisms of SGB for PSD

Melatonin is a neuroendocrine hormone synthesized and secreted by the pineal gland, and its secretion follows a circadian rhythm, with lower levels during the daytime and higher levels at night; hence, it is referred to as a physiological hypnotic.^[39]^ Melatonin binds to the melatonin receptors in the suprachiasmatic nucleus and plays an essential role in regulating the sleep–wake cycle.^[39]^ Surgery-induced trauma and blood can trigger stress responses, subsequently affecting the endocrine function of the body, leading to a severe disruption of the perioperative melatonin secretion rhythm.^[28,34]^ Melatonin therapy has an improving effect on sleep disorders related to circadian rhythm. The restoration of melatonin rhythm leads to the recovery of various physiological and biological rhythms, thereby preventing sleep–wave cycle disruption and sleep disorders caused by abnormal melatonin levels.^[39,40]^ Previous studies have indicated that SGB can increase melatonin secretion and reduce the release of inflammatory factors and cortisol, thereby improving the sleep quality and recovery quality of surgical patients.^[28,34]^ A clinical study in 2022 found that patients undergoing thoracoscopic lung cancer surgery commonly experience PSD.^[34]^ Compared to the control group, patients in the SGB group had longer durations of sleep at 24 and 48 hours postoperatively, higher sleep efficiency indices, and longer deep non-rapid eye movement sleep duration.^[34]^ Furthermore, patients in the SGB group had a lower Athens insomnia scale for subjective sleep quality postoperatively and a lower incidence of PSD.^[34]^ Additionally, patients in the SGB group had lower plasma concentrations of NE and cortisol postoperatively, as well as higher concentrations of 6-hydroxymelatonin in their morning urine. This study further demonstrates that SGB can improve early sleep quality in elderly lung cancer patients after surgery, thereby alleviating the vicious cycle between stress responses and sleep disorders and promoting better recovery.^[34]^ Perioperative psychological and physiological stress responses can lead to alternations in postoperative sleep architecture. Excessive secretion of corticotropin-releasing hormone can not only induce sleep disorders through the peripheral hypothalamic–pituitary–adrenal axis but also significantly suppress non-rapid eye movement sleep through the central pathway mediated by corticotropin-releasing hormone receptor.^[28,34]^ Under stress conditions, neurotrophic factors in the brain increase and are then transported to the SG, promoting the growth of neuronal synapses, and thereby increasing brain NE levels through reverse regulation by neural connections.^[26,41]^

Research has demonstrated that preoperative injection of local anesthetics around the SG can inhibit the production of neurotrophic factors, suppress synaptic growth, reduce NE levels, and decrease the release of inflammatory factors, thereby reducing PSD and pain in patients and accelerating recovery.^[42]^ Furthermore, SGB can block the excitatory conduction of the sympathetic nervous system, suppress stress responses, regulate the functions of the autonomic nervous and endocrine systems, thereby correcting the imbalance in neural function and hormone secretion and improving sleep quality.^[28]^ SGB can increase blood flow in the anastomotic artery between the temporal superficial artery and the middle cerebral artery after intracranial arterial bypass surgery.^[38]^ Additionally, SGB, by blocking the sympathetic nerve plexus around the vertebral and posterior cerebral arteries, reduces cerebral vascular resistance, increases cerebral blood flow, improves cerebral circulation, and promotes the recovery of cortical inhibitory processes in the brain, thereby achieving the goal of improving sleep.^[31,38,43]^ Surgical-induced inflammation plays a crucial role in the occurrence of PSD, and IL-1, IL-6, and TNF-α can increase slow wave sleep time while decreasing REM sleep time, which may be one of the reasons for short-term REM deprivation after surgery.^[28,34,44]^ In patients receiving SGB treatment, levels of C-reactive protein and IL-6 significantly decreased, indicating SGB can alleviate inflammation responses.^[21,28]^ Research confirmed that SGB can inhibit inflammatory and stress responses, reduce the occurrence of abdominal distension, shorten the time to pass gas, promote early recovery of gastrointestinal function, and decrease postoperative nausea and vomiting, all of which can alleviate PSD symptoms to some extent.^[9]^ Animal studies also indicate that continuous SGB treatment can improve learning and memory deficits in acute sleep-deprived rats, reducing the release of hippocampal inflammatory factors and neuronal apoptosis.^[45]^ Similarly, SGB improves postoperative cognitive function in elderly rats by downregulating hippocampal adenosine-activated protein kinase and inhibiting the activation of astrocytes.^[45]^ Furthermore, SGB treatment increases the expression levels of SIRT1 in the hippocampus and white matter while reducing the activity of NF-κB in both areas, further improving cognitive deficits, neuroinflammation, and neuronal apoptosis in the white matter.^[46]^

## 7. Limitation of SGB and future perspective of SGB research

Likewise, SGB is beneficial in the treatment of anxiety symptoms from post-traumatic stress disorder. Even though, more clinical studies with larger sample sizes and alternate protocols are needed to further explore the therapeutic potential of SGB. After the procedure, patients are advised to stay in the clinic for a brief period of monitoring. They may experience minor, temporary side effects such as Horner syndrome, which can include a droopy eyelid, redness in the treated eye, or a hoarse voice. These effects are normal and indicate that the procedure was successful; they typically resolve within 4 to 8 hours. Other mild side effects, such as nasal congestion, headache, or slight soreness and swelling at the injection site, may occur but are easily manageable.

In the presence of the advancement of ultrasound technology, SGB has been increasingly applied across various clinical disciplines. In addition to the advancement in clinical settings, research on SGB utilizing animal models has also shown a promising future. There is, however, a need for further studies to delve deeper into understanding the potential mechanism of action of SGB across various diseases, as shown in Table 3.^[25,45–50]^ Moreover, as a regional blockade method, SGB offers a versatile approach to treating pain and a wide range of conditions, characterized by minimal side effects. We have applied the bulimorexia analysis that found that SGB plays a pivotal role in improving perioperative PSD by balancing the secretion of melatonin, suppressing perioperative stress responses, enhancing cerebral blood flow, and inhibiting the release of inflammatory factors.^[14]^ Furthermore, SGB contributes to a reduction in the incidence of perioperative complications and expedites postoperative recovery, representing a novel perioperative rehabilitation model in recent years. While SGB exhibits great promise for the treatment of PSD, it can also call for further research, particularly in the development of standardized treatment protocols, exploration of related mechanisms, investigation into molecular mechanisms, and comparisons with medication therapies in the context of PSD treatment. The stellate ganglion, a component of the sympathetic nervous system, is a cluster of nerves situated between the sixth (C6) and seventh (C7) cervical vertebrae. SGB is an outpatient procedure in which clinicians inject a local anesthetic, such as ropivacaine 0.5%, into the stellate ganglion. This injection inhibits nerve impulses to the head, neck, and upper extremities.^[51]^ Given that the stellate ganglion is connected to the amygdala, a brain region believed to be abnormally activated in PTSD-SGB has been explored as a potential treatment for PTSD. However, the exact mechanism by which SGB alleviates PTSD symptoms remains unclear. Additionally, SGB has been assessed for its potential benefits in treating anxiety and depression.^[52]^ Traditional PTSD treatments typically involve psychotherapy and/or pharmacotherapy. Various forms of psychotherapy, such as exposure-based therapy, cognitive-behavioral therapy, and stress inoculation training, are commonly used.^[51]^ Pharmacological treatments may include antidepressants (e.g., selective serotonin reuptake inhibitors, serotonin-NE reuptake inhibitors), antipsychotic drugs, mood stabilizers, and other agents.^[51]^ However, the success rates of these treatments are generally variable, with remission rates ranging from 30% to 40%. As a result, alternative therapies like SGB are being evaluated for their potential effectiveness in treating PTSD.^[15]^

**Table 3:** Examples of applications of the stellate ganglion block for various animal models.

Study	SGB	Model	Test	Outcomes
Dai et al^[47]^	0.2% right site bupivacaine 0.2 mL	SD rat sleep deprivation for 4 days	① WM ② ELISA ③ WB	① The expression of IL-6, IL-1β and Caspase-3 in the hippocampus was decreased. ② Rat serum melatonin was also increased. ③ SGB can improve the spatial learning and memory dysfunction of rats with sleep deprivation.
Bi et al^[45]^	0.25% right site bupivacaine 0.15 mL	SGB + splenectomy	① WM ② PCR ③ WB	SGB can reduce the POCD, and the mechanism may be associated with downregulating the expression of AMPK and restraining the activation of the astrocyte.
Hao et al^[48]^	0.25% right site bupivacaine 0.15 mL	SGB + celiac exploration	① WM ② Immunohistochemical ③ WB	SGB increases Bcl-2 expression in the rat hippocampus, decreases Bax expression, suppresses neuronal apoptosis, and reduces POCD
Zhao et al^[49]^	0.25% right site bupivacaine 0.15 mL	SGB + celiac exploration	① WM ② Immunohistochemical ③ WB	SGB recued the POCD in aged rats, and the mechanism may be related to the inhibition of phosphorylated tau expression in hippocampal tissue
Chen et al^[50]^	0.25% right site bupivacaine 0.15 mL	SGB + celiac exploration	① WM ② Immunohistochemical ③ WB	SGB can slow down the increase of serum S100 β and NSE concentration in old rats and improve the postoperative brain histopathological changes
Shi et al^[25]^	0.2 mL of 0.25% ropivacaine	Central poststroke pain model	① OFT ② EPMT ③ NSFT ④ FST ⑤ Immunofluorescence ⑥ WB ⑦ ELISA	SGB ameliorated central poststroke pain with comorbid anxiety and depression through inhibiting HIF-1α/NLRP3 signaling following
Zhang et al^[46]^	–	SGB + splenectomy	WM Immunohistochemistry WB ELISA	SGB ameliorates cognitive impairment, neuroinflammation and neuronal apoptosis in white matter. SGB enhanced the expression of SIRT1 in the hippocampus and white matter, decreased NF-κB activity in the hippocampus and white matter.

ELISA = enzyme-linked immunosorbent assay, EPMT = elevated plus maze test, FST = forced swim test, NSFT = novelty-suppressed feeding test, OFT = open field test, PCR = polymerase chain reaction, POCD = postoperative cognitive dysfunction, SD = sleep deprivation, SGB = stellate ganglion block, WB = western blotting, WM = water maze.

## 8. Conclusion

In summary, the SGB shows promise in treating PSD by modulating sympathetic nervous system activity. This review highlights the anatomical basis, clinical applications, and potential mechanisms of SGB. While SGB has demonstrated benefits in improving sleep quality and reducing PSD incidence, further research is needed to standardize techniques and elucidate underlying mechanisms. SGB is a valuable intervention for enhancing postoperative recovery and warrants continued exploration.

## Author contributions

**Conceptualization:** Jianghui Luo, Xudong Zhang, Xiaohong Tan, Jiazun Chen, Miaomiao Tang.

**Data curation:** Xudong Zhang, Jiazun Chen, Miaomiao Tang.

**Formal analysis:** Jianghui Luo, Xudong Zhang, Xiaohong Tan, Miaomiao Tang.

**Investigation:** Jianghui Luo, Xudong Zhang, Jiazun Chen, Miaomiao Tang.

**Methodology:** Jianghui Luo, Xudong Zhang, Xiaohong Tan, Jiazun Chen.

**Project administration:** Jianghui Luo, Xudong Zhang, Xiaohong Tan, Jiazun Chen, Miaomiao Tang.

**Resources:** Xudong Zhang.

**Software:** Xudong Zhang, Jiazun Chen, Miaomiao Tang.

**Supervision:** Jianghui Luo, Xudong Zhang, Xiaohong Tan, Miaomiao Tang.

**Validation:** Jianghui Luo, Xudong Zhang, Xiaohong Tan, Miaomiao Tang.

**Visualization:** Jianghui Luo, Xudong Zhang, Xiaohong Tan, Jiazun Chen, Miaomiao Tang.

**Writing – original draft:** Jianghui Luo, Xudong Zhang, Xiaohong Tan, Jiazun Chen, Miaomiao Tang.

**Writing – review & editing:** Jianghui Luo, Xudong Zhang, Xiaohong Tan, Jiazun Chen, Miaomiao Tang.

## References

[1] Abdi S Yang Z. A novel technique for experimental stellate ganglion block in rats. Anesth Analg. 2005;101:561–5. 16037176 10.1213/01.ANE.0000159169.12425.50

[2] Jeong S Jeon Y Yeo J Baek W. The effects of stellate ganglion block on the electroencephalogram in rats. J Anesth. 2014;28:601–5. 24408532 10.1007/s00540-013-1780-8

[3] Singh H Rajarathinam M. Stellate ganglion block beyond chronic pain: a literature review on its application in painful and non-painful conditions. J Anaesthesiol Clin Pharmacol. 2024;40:185–91. 38919437 10.4103/joacp.joacp_304_22 PMC11196062

[4] Hua L Sha K Lu H. Clinical efficacy evaluation of ultrasound-guided C2 dorsal root nerve pulsed radiofrequency combined with stellate ganglion block in the treatment of cervicogenic headache: a retrospective cohort study. J Pain Res. 2023;16:2655–63. 37533562 10.2147/JPR.S409226 PMC10392809

[5] Narouze S. Ultrasound-guided stellate ganglion block: safety and efficacy. Curr Pain Headache Rep. 2014;18:424. 24760493 10.1007/s11916-014-0424-5

[6] Wittwer ED Radosevich MA Ritter M Cha YM. Stellate ganglion blockade for refractory ventricular arrhythmias: implications of ultrasound-guided technique and review of the evidence. J Cardiothorac Vasc Anesth. 2020;34:2245–52. 31919004 10.1053/j.jvca.2019.12.015

[7] Rae Olmsted KL Bartoszek M Mulvaney S. Effect of stellate ganglion block treatment on posttraumatic stress disorder symptoms: a randomized clinical trial [published correction appears in JAMA Psychiatry. 2020;77:218. doi: 10.1001/jamapsychiatry.2019.4511] 2020 Sep 1;77(9):982. doi: 10.1001/jamapsychiatry.2020.1829]. JAMA Psychiatry. 10.1001/jamapsychiatry.2019.3474 PMC6865253 31693083

[8] Lipov EG Joshi JR Xie H Slavin KV. Updated findings on the effects of stellate-ganglion block on hot flushes and night awakenings. Lancet Oncol. 2008;9:819–20. 18760240 10.1016/S1470-2045(08)70218-3

[9] Zhu G Kang Z Chen Y Zeng J Su C Li S. Ultrasound-guided stellate ganglion block alleviates stress responses and promotes recovery of gastrointestinal function in patients. Dig Liver Dis. 2021;53:581–6. 33303314 10.1016/j.dld.2020.11.028

[10] Lin SZ Chen L Tang YJ. Establishment of ultrasound-guided stellate ganglion block in rats. Front Neurosci. 2023;16:1061767. 36711146 10.3389/fnins.2022.1061767 PMC9877532

[11] Huntoon MA. The vertebral artery is unlikely to be the sole source of vascular complications occurring during stellate ganglion block. Pain Pract. 2010;10:25–30. 19761512 10.1111/j.1533-2500.2009.00310.x

[12] Martinez FA. The anatomy of the stellate ganglion and its surgical approach. Bull Georgetown Univ Med Cent. 1954;7:130–5. 13140994

[13] Guttuso T Jr. Stellate ganglion block for treating hot flashes: a viable treatment option or sham procedure? Maturitas. 2013;76:221–4. 24021996 10.1016/j.maturitas.2013.08.001

[14] Yan S Wang Y Yu L. Stellate ganglion block alleviates postoperative sleep disturbance in patients undergoing radical surgery for gastrointestinal malignancies. J Clin Sleep Med. 2023;19:1633–42. 37128727 10.5664/jcsm.10632 PMC10476041

[15] Lynch JH Mulvaney SW Kim EH de Leeuw JB Schroeder MJ Kane SF. Effect of stellate ganglion block on specific symptom clusters for treatment of post-traumatic stress disorder. Mil Med. 2016;181:1135–41. 27612365 10.7205/MILMED-D-15-00518

[16] Lipov EG Lipov S Joshi JR Santucci VD Slavin KV Beck Vigue SG. Stellate ganglion block may relieve hot flashes by interrupting the sympathetic nervous system. Med Hypotheses. 2007;69:758–63. 17425958 10.1016/j.mehy.2007.01.082

[17] Liao CD Tsauo JY Chen HC Liou TH. Efficacy of stellate ganglion blockade applied with light irradiation: a systemic review and meta-analysis. Am J Phys Med Rehabil. 2017;96:e97–e110. 28118275 10.1097/PHM.0000000000000675

[18] Hao W Yang R Yang Y. Stellate ganglion block ameliorates vascular calcification by inhibiting endoplasmic reticulum stress. Life Sci. 2018;193:1–8. 29208463 10.1016/j.lfs.2017.12.002

[19] Chen YQ Xie YY Wang B Jin XJ. Effect of stellate ganglion block on hemodynamics and stress responses during CO_2_-pneumoperitoneum in elderly patients. J Clin Anesth. 2017;37:149–53. 28235510 10.1016/j.jclinane.2016.12.003

[20] Yildirim V Akay HT Bingol H. Pre-emptive stellate ganglion block increases the patency of radial artery grafts in coronary artery bypass surgery. Acta Anaesthesiol Scand. 2007;51:434–40. 17378781 10.1111/j.1399-6576.2006.01260.x

[21] Lipov E Gluncic V Lukić IK Candido K. How does stellate ganglion block alleviate immunologically-linked disorders? Med Hypotheses. 2020;144:110000. 32758866 10.1016/j.mehy.2020.110000

[22] Li Y Du HB Jiang LN. Stellate ganglion block improves the proliferation and function of splenic CD4 + T cells through inhibition of posthemorrhagic shock mesenteric lymph-mediated autophagy. Inflammation. 2021;44:2543–53. 34533673 10.1007/s10753-021-01523-x

[23] Wang QX Wang XY Fu NA Liu JY Yao SL. Stellate ganglion block inhibits formalin-induced nociceptive responses: mechanism of action. Eur J Anaesthesiol. 2005;22:913–8. 16318661 10.1017/S0265021505001559

[24] Jeon Y. Therapeutic potential of stellate ganglion block in orofacial pain: a mini review. J Dent Anesth Pain Med. 2016;16:159–63. 28884148 10.17245/jdapm.2016.16.3.159 PMC5586552

[25] Shi ZM Jing JJ Xue ZJ. Stellate ganglion block ameliorated central post-stroke pain with comorbid anxiety and depression through inhibiting HIF-1α/NLRP3 signaling following thalamic hemorrhagic stroke. J Neuroinflammation. 2023;20:82. 36944982 10.1186/s12974-023-02765-2 PMC10031944

[26] Bajor LA Balsara C Osser DN. An evidence-based approach to psychopharmacology for posttraumatic stress disorder (PTSD) – 2022 update. Psychiatry Res. 2022;317:114840. 36162349 10.1016/j.psychres.2022.114840

[27] Yang X Wu Q Wang H Zhang Y Peng X Chen L. Effects of ultrasound-guided stellate ganglion block on postoperative quality of recovery in patients undergoing breast cancer surgery: a randomized controlled clinical trial. J Healthc Eng. 2022;2022:7628183. 36046011 10.1155/2022/7628183 PMC9424037

[28] Yang RZ Li YZ Liang M. Stellate ganglion block improves postoperative sleep quality and analgesia in patients with breast cancer: a randomized controlled trial. Pain Ther. 2023;12:491–503. 36652140 10.1007/s40122-022-00473-y PMC10036705

[29] Xu J Liu Q Huang T Zhong R Zhang Y. Stellate ganglion block rectifies excessive daytime sleepiness: a case report. J Int Med Res. 2022;50:3000605221118681. 35983675 10.1177/03000605221118681 PMC9393672

[30] Wu CN Wu XH Yu DN Ma WH Shen CH Cao Y. A single-dose of stellate ganglion block for the prevention of postoperative dysrhythmias in patients undergoing thoracoscopic surgery for cancer: a randomised controlled double-blind trial. Eur J Anaesthesiol. 2020;37:323–31. 31860606 10.1097/EJA.0000000000001137

[31] Rahimzadeh P Imani F Nafissi N Ebrahimi B Faiz SHR. Comparison of the effects of stellate ganglion block and paroxetine on hot flashes and sleep disturbance in breast cancer survivors. Cancer Manag Res. 2018;10:4831–7. 30464591 10.2147/CMAR.S173511 PMC6208490

[32] Lipov EG Joshi JR Sanders S. Effects of stellate-ganglion block on hot flushes and night awakenings in survivors of breast cancer: a pilot study. Lancet Oncol. 2008;9:523–32. 18485819 10.1016/S1470-2045(08)70131-1

[33] Haest K Kumar A Van Calster B. Stellate ganglion block for the management of hot flashes and sleep disturbances in breast cancer survivors: an uncontrolled experimental study with 24 weeks of follow-up. Ann Oncol. 2012;23:1449–54. 22039079 10.1093/annonc/mdr478

[34] Gu C Zhai M Lü A. Ultrasound-guided stellate ganglion block improves sleep quality in elderly patients early after thoracoscopic surgery for lung cancer: a randomized controlled study. Nan Fang Yi Ke Da Xue Xue Bao. 2022;42:1807–14. 36651248 10.12122/j.issn.1673-4254.2022.12.08 PMC9878408

[35] Geng J Wang J Zhang Y. The effect of a combined modified pectoral and stellate ganglion block on stress and inflammatory response in patients undergoing modified radical mastectomy. Int J Breast Cancer. 2022;2022:3359130. 35707316 10.1155/2022/3359130 PMC9192316

[36] Chouchou F Khoury S Chauny JM Denis R Lavigne GJ. Postoperative sleep disruptions: a potential catalyst of acute pain? Sleep Med Rev. 2014;18:273–82. 24074687 10.1016/j.smrv.2013.07.002

[37] Kain ZN Caldwell-Andrews AA. Sleeping characteristics of adults undergoing outpatient elective surgery: a cohort study. J Clin Anesth. 2003;15:505–9. 14698361 10.1016/j.jclinane.2003.02.002

[38] Kang CK Oh ST Chung RK. Effect of stellate ganglion block on the cerebrovascular system: magnetic resonance angiography study. Anesthesiology. 2010;113:936–44. 20823762 10.1097/ALN.0b013e3181ec63f5

[39] Pandi-Perumal SR Trakht I Spence DW Srinivasan V Dagan Y Cardinali DP. The roles of melatonin and light in the pathophysiology and treatment of circadian rhythm sleep disorders. Nat Clin Pract Neurol. 2008;4:436–47. 18628753 10.1038/ncpneuro0847

[40] Uchida K Tateda T Hino H. Novel mechanism of action hypothesized for stellate ganglion block related to melatonin. Med Hypotheses. 2002;59:446–9. 12208186 10.1016/s0306-9877(02)00158-5

[41] Lipov E Ritchie EC. A review of the use of stellate ganglion block in the treatment of PTSD. Curr Psychiatry Rep. 2015;17:599. 26073361 10.1007/s11920-015-0599-4

[42] Takatori M Kuroda Y Hirose M. Local anesthetics suppress nerve growth factor-mediated neurite outgrowth by inhibition of tyrosine kinase activity of TrkA. Anesth Analg. 2006;102:462–7. 16428543 10.1213/01.ane.0000194334.69103.50

[43] Gupta MM Bithal PK Dash HH Chaturvedi A Mahajan RP. Effects of stellate ganglion block on cerebral haemodynamics as assessed by transcranial Doppler ultrasonography. Br J Anaesth. 2005;95:669–73. 16155036 10.1093/bja/aei230

[44] Bryant PA Trinder J Curtis N. Sick and tired: does sleep have a vital role in the immune system? Nat Rev Immunol. 2004;4:457–67. 15173834 10.1038/nri1369

[45] Bi Y Wang B Yin Z Zhang G Chen H Wang M. Effects of stellate ganglion block on AMP-activated protein kinase and astrocyte in hippocampal neurones in postoperative aged rats. Zhonghua Yi Xue Za Zhi. 2014;94:2222–6. 25331477

[46] Zhang J Liu Y Li H. Stellate ganglion block improves postoperative cognitive dysfunction in aged rats by SIRT1-mediated white matter lesion repair. Neurochem Res. 2022;47:3838–53. 36315371 10.1007/s11064-022-03800-z

[47] Dai D Zheng B Yu Z. Right stellate ganglion block improves learning and memory dysfunction and hippocampal injury in rats with sleep deprivation. BMC Anesthesiol. 2021;21:272. 34749669 10.1186/s12871-021-01486-4 PMC8574040

[48] Hao J Wenbo C Xiaobing L. Effect of stellate ganglion block on Bcl-2 and Bax expression and postoperative cognitive function in the hippocampus of aged rats. Shaanxi J Med. 2019;48:1422–5.

[49] Zhao Y Huanglong Yanan D. Effects of stellate ganglion block on postoperative cognitive function in aged rats. Chin J Clin (electronic edition). 2015;9:1654–7.

[50] Chen Y Xiaohong D Xia J. Effect of stellate ganglion block on serum S100 β protein, NSE, and postoperative cognitive function in aged rats. J Clin Anesthesiol. 2013;29:1020–3.

[51] Peterson K Bourne D Anderson J Mackey K Helfand M. Evidence Brief: Effectiveness of Stellate Ganglion Block for Treatment of Posttraumatic Stress Disorder (PTSD). Department of Veterans Affairs (US); 2017. 28742302

[52] Hanling SR Hickey A Lesnik I. Stellate ganglion block for the treatment of posttraumatic stress disorder: a randomized, double-blind, controlled trial. Reg Anesth Pain Med. 2016;41:494–500. 27187898 10.1097/AAP.0000000000000402

